# Taxon ordering in phylogenetic trees: a workbench test

**DOI:** 10.1186/1471-2105-12-58

**Published:** 2011-02-22

**Authors:** Francesco Cerutti, Luigi Bertolotti, Tony L Goldberg, Mario Giacobini

**Affiliations:** 1 Department of Animal Production, Epidemiology and Ecology, Faculty of Veterinary Medicine, University of Torino, via Leonardo da Vinci 44, 10095, Grugliasco (TO), Italy; 2 Molecular Biotechnology Center, University of Torino, via Nizza 52, 10126, Torino, Italy; 3 Department of Pathobiological Sciences, School of Veterinary Medicine, University of Wisconsin-Madison, 1656 Linden Drive, Madison, Wisconsin, 53706, USA

## Abstract

**Background:**

Phylogenetic trees are an important tool for representing evolutionary relationships among organisms. In a phylogram or chronogram, the ordering of taxa is not considered meaningful, since complete topological information is given by the branching order and length of the branches, which are represented in the root-to-node direction. We apply a novel method based on a (λ + *μ*)-Evolutionary Algorithm to give meaning to the order of taxa in a phylogeny. This method applies random swaps between two taxa connected to the same node, without changing the topology of the tree. The evaluation of a new tree is based on different distance matrices, representing non-phylogenetic information such as other types of genetic distance, geographic distance, or combinations of these. To test our method we use published trees of Vesicular stomatitis virus, West Nile virus and Rice yellow mottle virus.

**Results:**

Best results were obtained when taxa were reordered using geographic information. Information supporting phylogeographic analysis was recovered in the optimized tree, as evidenced by clustering of geographically close samples. Improving the trees using a separate genetic distance matrix altered the ordering of taxa, but not topology, moving the longest branches to the extremities, as would be expected since they are the most divergent lineages. Improved representations of genetic and geographic relationships between samples were also obtained when merged matrices (genetic and geographic information in one matrix) were used.

**Conclusions:**

Our innovative method makes phylogenetic trees easier to interpret, adding meaning to the taxon order and helping to prevent misinterpretations.

## Background

Phylogenetic trees are an important tool in evolutionary biology for representing the history of evolution of organisms. They are composed of nodes, representing hypothetical ancestors, and branches or edges, reflecting the relationship between nodes. Terminal nodes or taxa represent the taxa whose evolution has been investigated, and they can represent extant or extinct organisms [1]. In phylograms and chronograms, branches contain information, e.g. character changes or evolutionary time; in both cases they represent distances between nodes. Consequently, the taxon order is meaningless, and the closeness of taxa can be misleading. For example, two taxa lying adjacent to each other on a phylogenetic tree may actually be very distantly related, creating problems of interpretation.

Previous work has already accepted the challenge of ordering the taxa to add a meaning according to the genetic distance. The software Neighbor-Net [2] is shown to build a network which also minimizes the distance among taxa, given a matrix that fulfill the Kalmanson inequalities [3,4]. This software relies on phylogenetic networks, using an algorithm based on Neighbor Joining [5]. Levy and Pachter [4] showed that the algorithm, considering the problem as a "traveling salesman" problem, is robust for ordering taxa according to a distance matrix. Other studies have endeavored to minimize the distance between taxa as a minimum Hamiltonian path [6,7], either while building the tree or after having built it.

**Figure 1:**
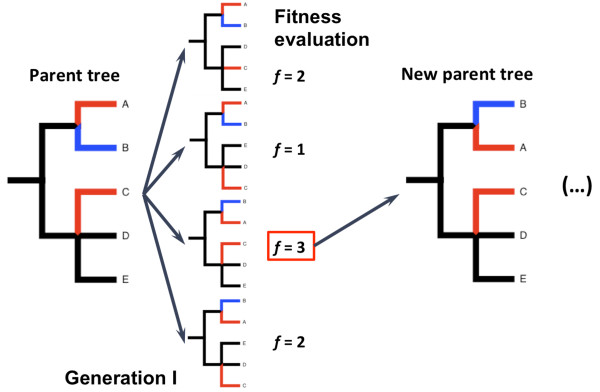
**Evolutionary algorithm**. Starting from the parent tree, the algorithm generates possible solutions randomly rotating branches connected to the same node. After the fitness evaluation, the best individuals are selected as the parent population in the next cycle of the algorithm.

In our previous work [8,9] we introduced an innovative method to order taxa on a phylogenetic tree to make taxon order more meaningful. We used (λ + *μ*) - Evolutionary Algorithms (EAs) [10,11] to reorganize taxon order by random rotation of internal nodes (thus not modifying the topology), evaluating the modified tree on the basis of genetic distances. Figure 1 reports a brief scheme of the process to generate and select the optimal tree. The main goal is to order the taxa on a phylogenetic tree according to any given distance matrix. The tree can be built with any software for phylogenetic inference and then used as input for our algorithm.

In the current study we apply this method to different data sets using different types of distances. We chose published phylogenetic trees of three RNA viruses infecting different hosts with different modes of transmission: Vesicular stomatitis virus (VSV) presented by Perez et al. [12], West Nile virus (WNV) presented by Bertolotti et al. [13] and Rice yellow mottle virus (RYMV) presented by Abubakar et al. [14]. We reorganized each tree using genetic and geographic distance matrices as well as combined distances (geographic and genetic) to improve graphical representation by optimizing the order of taxa.

## Results and discussion

### VSV case study

**Figure 2:**
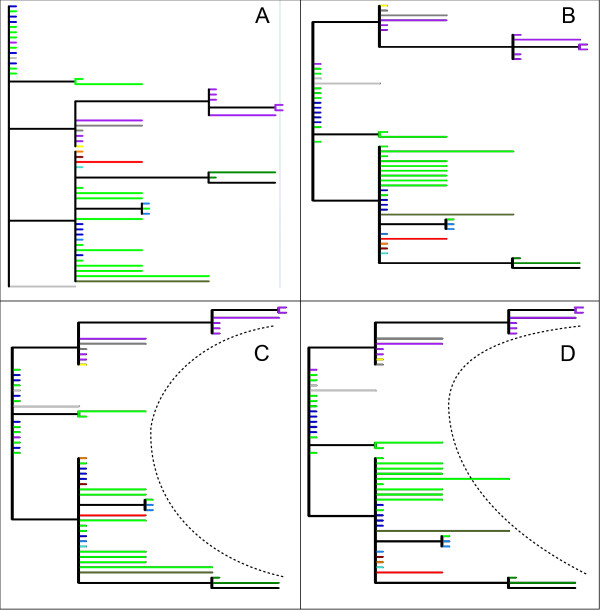
**VSV trees**. The original tree, as presented by Perez et al. (A). The best trees obtained using the geographic (B), genetic (C) and combined (D) distances. The dashed line in C and D highlights the "C" shape acquired by the clades.

**Figure 3:**
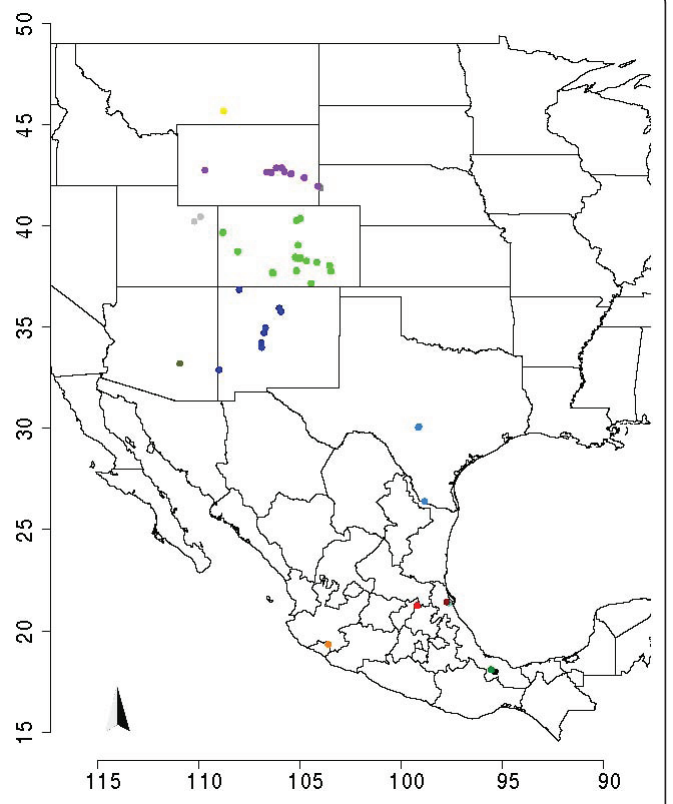
**Map of VSV samples**. Map representing the study area of USA and Mexico where VSV samples were collected. Each site has a color, that is the same of the tips of the samples collected in it.

VSV is a negative-sense single-stranded RNA virus member of the family *Rhabdoviridae*, which causes vesicular stomatitis in horses, cattle, swine, and certain wildlife species [15]. Starting from the tree originally published in [12], we created an Euclidean distance matrix from collection sites coordinates. Then, the EA was run 50 times, each starting with the original tree plus λ-1 different initial trees (see Methods), generated from the original one (Figure 2a). The performance of the runs was comparable, and we analyzed the best trees (Figure 2b). The order of taxa follows a clear north-south progression, reflecting the geographic arrangement of collection sites, represented in the map in Figure 3. In other words, the algorithm was able to group those taxa belonging to the same state (geographically closer each other). We also tested the algorithm using a separate genetic distance matrix and a combined genetic and geographic distance matrix. Using genetic distances only, the improvement in tree representation is less evident, as might be expected considering that the original tree was constructed using genetic data. However, the EA returns a tree in which the most genetically divergent clades are moved to the extremities of the phylogeny, leading to a "C"-like shape, as shown in Figure 2c. The effect of combining the two matrices is strongly evident: the tree has the same "C"-like shape arrangement as the genetically modified tree, and moreover it conserves the aggregation of taxa from same states (locations) (Figure 2d).

### WNV case study

**Figure 4:**
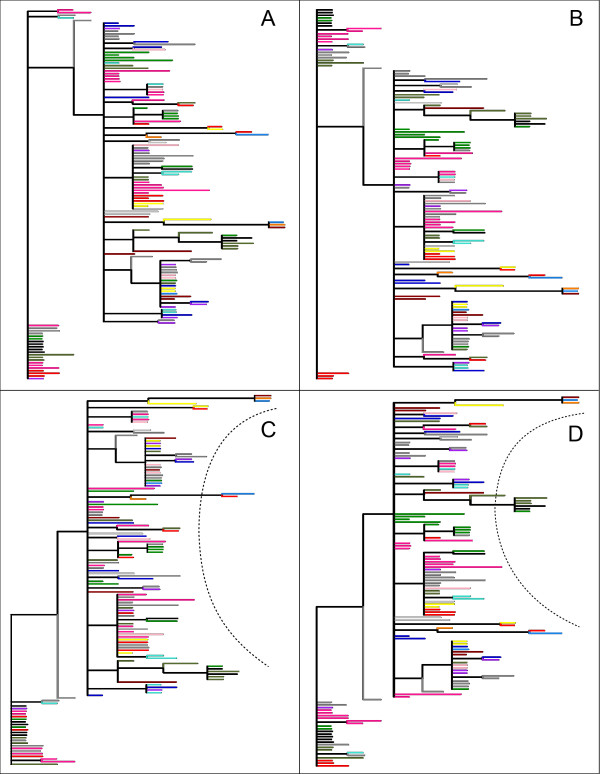
**WNV trees**. The original tree, as presented by Bertolotti et al. (A). The best trees obtained using the geographic (B), genetic (C) and combined (D) distances. The dashed line in C and D highlights the "C" shape acquired by the clades.

**Figure 5:**
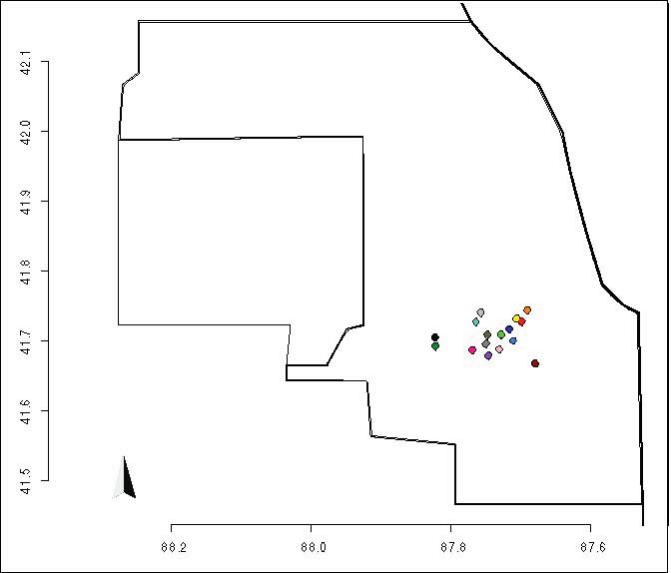
**Map of WNV samples**. Map representing Cook and DuPage counties, and the collection sites used in [30]. Each site has a color, that is the same of the tips of the samples collected in it.

WNV is a positive-sense single-stranded RNA virus belonging to the family *Flaviviridae*, and it is transmitted primarily through the bite of infected mosquitoes to birds. Occasionally it infects horses and humans causing West Nile febrile illness and neurologic disease [16]. The original tree, from [13], shown in Figure 4a, was rearranged using a matrix of geographic distances, as described in the Methods section. In the case of WNV, the geographic arrangement of locations is not linear (Figure 5), as in the case of VSV. In the modified tree (Figure 4b), taxa nevertheless group by sampling location. Due to large sample size and constraints of tree topology, this grouping is less evident than in case of VSV.

In the original paper, the authors declared that more than the 90% of viral genetic variance was contained within sampling sites. Modifying the tree using a matrix of genetic distances yielded a tree that did not tend to group taxa by geographic location (Figure 4c); rather, the modified tree acquired the aforementioned "C"-like shape. As reported in the same paper, the authors found a significant association between viral genetic and spatial distances, with samples collected from the same site likely to be genetically similar. The modified tree obtained with merged genetic and geographic distance matrices tended to move samples collected from the same location closer, supporting the original contention that that viruses from the same location are also genetically similar (Figure 4d). Furthermore the "C"-like shape of the tree was conserved even when merged matrices were used. The new trees obtained therefore graphically support the statistical analyses in the original publication showing weak genetic-geographic association [13].

### RYMV case study

**Figure 6:**
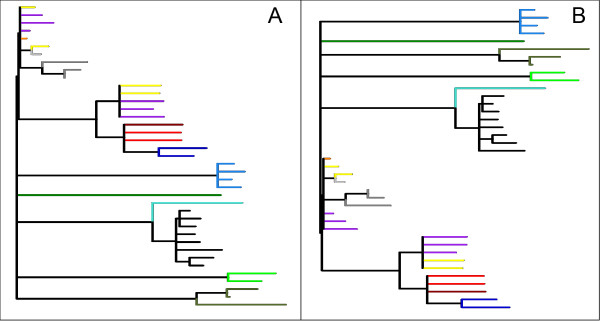
**RYMV trees**. The original tree, as presented by Abubakar et al. (A). The best trees obtained using the geographic (B) distances.

RYMV is a positive-sense single-stranded RNA virus belonging to the genus Sobemovirus and it is considered to be among the most important rice pathogens in sub-Saharan Africa [17,18]. The original tree of RYMV, published in [14], is shown in Figure 6a and has fewer taxa than trees in the previous examples. For this reason, RYMV provided a convenient system to examine the influence of the *radius *parameter in the fitness evaluation, where we previously showed [8] that *r *= 8 offers an acceptable balance between computational intensity and accuracy. In the RYMV case, where the tree had 39 terminal taxa, *r *= 8 was too large to discriminate differences among samples, such that we examined *r *∈ {2, 4}. The best relative fitness improvement was obtained by *r *= 2, since this value was able to discriminate well among distances in the process of fitness evaluation.

**Figure 7:**
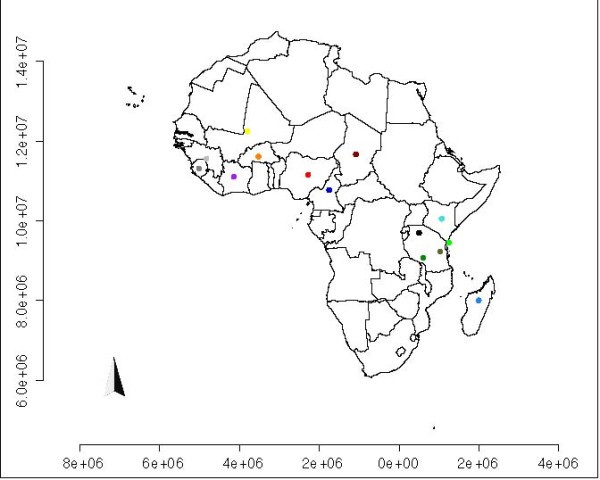
**Map of RYMV samples**. Map representing African states and sample origins, as reported in [14]. Each site has a color, that is the same of the tips of the samples collected in it.

The tree modified using geographic distance (shown in Figure 6b) shows two clusters of geographic locations: samples from West and East Africa (see map in Figure 7). Within clusters, the taxon order reflects the geographic relationship on the map. In addition, samples from central Africa (dots in red, dark red and dark blue on the map) were moved farther away from Tanzanian samples (dots in greens and black) than from samples in Western African countries. This result may be explained by the fact that the algorithm would be expected to optimize taxon order within clusters more efficiently than between clusters. We note that manually modifying the tree in order to improve geographic order often returned a worse fitness compared to the artificially evolved tree (data not shown).

## Conclusions

In our previous studies [8,9] we introduced an innovative method to give a meaning to the order of taxa on a phylogenetic tree using an evolutionary algorithm. Here we test the algorithm using different viral systems, trees and distances with the aim of improving the graphic representation of the tree without modifying its topology. Qualitatively, the most improved tree was obtained when we evolved the VSV phylogenetic tree using a matrix of geographic distances, since the order of taxa on the improved tree closely mirrored geography. In the case of WNV, our algorithm generated trees that support previous studies of genetic diversity and spatial correlation. In the case of RYMV, we identified a possible limitation of the algorithm: the improvement in graphical representation may be less pronounced when the tree contains small numbers of taxa. For this reason we evaluated the role of *r*, highlighting that, in case of small sample sets, small radii may be sufficient to achieve marked improvements. The strength of this method is that a phylogeny can be inferred using any available method for building trees, using distance and algorithm, from a simple approach (like Neighbor-Joining) to a more accurate Bayesian inference. The user can choose the appropriate method, using the genetic information contained in the considered sequences and, in the next step, add a second matrix, containing for example geographic information. The tests of the algorithm presented here showed promise. Very good results were achieved when the geographic distribution of the samples was linear, as in the VSV and RYMV cases. When the geographic distribution was more complex, as in the case of WNV, the grouping of taxa collected from the same site is hard to see. In this case, geographic information alone is not enough, but much greater improvement is gained when both genetic and geographic information are incorporated, as shown in Figure 3d. In conclusion, the algorithm tested in this paper offers a customizable method that can help biologists to better represent results of their phylogenetic analyses, improving the interpretation of phylogenetic trees and making them more understandable.

## Methods

### (5 + 5)- EA

The (5 + 5)- EA used in the present study is a particular case of the large family of (λ + *μ*)-EAs. As briefly reported in Figure 1, the algorithm starts from an original tree and creates. λ-1 trees by random swaps of pairs of internal nodes. In this way, a total of λ starting trees, with the same topology but different order of taxa, are obtained. In each generation, *μ *trees are generated by random mutation of those selected with *μ *tournaments between couples chosen by random sampling with reintroduction among the λ trees. The next generation parents are then the λ fittest trees among the λ + *μ *ones.

The selection of the best trees is based on the fitness evaluated as the sum of the distances between each taxon and the next *r *tips, where *r *is the radius in the fitness evaluation. The distances are contained in a matrix, used as input for the algorithm. Any available data that are able to discriminate among taxa can be used to generate the distance matrix. As a starting tree, an original tree obtained by any available method of phylogenetic inference with any method (e.g. distance-based, parsimony-based, or likelihood-based) is suitable.

### Experimental validations

For the experimental validation of the algorithm, we selected three phylogenetic trees from published works, following two criteria: availability of genetic and geographic data and published phylogenetic trees and associated phylogeographic interpretations. All the distance matrices were normalized to the highest value in order to have values lying in the range [0, 1].

#### VSV data

We reconstructed the tree presented by Perez et al. using the maximum-likelihood optimality criterion as implemented in PAUP* version b10 [19] and the nucleotide substitution parameters as estimated using Modeltest, version 3.7 [20], as reported in [12]. Unlike the original tree, the new tree contains only 55 taxa instead of 59, because of availability of both sequences and geographic coordinates. For the genetic distance matrix, we used nucleotide-level distances corrected with the HKY substitution model [21]. For the geographic distance matrix, we used euclidean distances between collection sites in a Latitude/Longitude coordinate system. The combined matrix of distances was created averaging the cells in the genetic and geographic matrices.

#### WNV data

The tree is from Bertolotti et al. [13] and contains 140 samples collected from Chicago, USA. We re-evaluated the best evolution model with Modeltest, version 3.7, and inferred the phylogeny using MrBayes software [22,23]. The genetic distance matrix was created from nucleotide sequence data and an uncorrected p-distance [24]. The geographic distance matrix was created from Euclidean distances between collection sites in a Latitude/Longitude coordinate system. The combined matrix of distances was created with the same method used for the VSV example.

#### RYMV data

RYMV is a positive-sense single-stranded RNA virus belonging to the genus Sobemovirus and it is considered to be among the most important rice pathogens within sub-Saharan Africa [17,18]. We considered the tree published by Abubakar et al. and re-built it using neighbor-joining tree and pairwise nucleotide sequence distances with the Kimura two-parameters model, as reported in the original paper [14], but removing the out-group sequence; thus the tree has 39 taxa instead of 40. The geographic distances were computed as a Euclidean distances between the centroids of the district (for Tanzania) or of the area of the state (for the other states) in a UTM coordinate system, zone 36.

### Geographic data

Data on country borders were obtained from shapefiles available online, managed and plotted with R software [25] and the packages *maptools *[26] and *shape *[27]. In particular, USA border data were downloaded from http://www.census.gov/geo/www/cob/st2000.html in ESRI Shapefile (.shp) format for all 50 States, D.C., and Puerto Rico; Mexico shapefile was downloaded from http://www.vdstech.com/map_data.htm and Africa shapefile was downloaded from http://www.maplibrary.org/index.php. Collection site coordinates for VSV were kindly provided by L. Rodriguez, A. Perez and S. Pauszek. WNV collection site coordinates were already available. RYMV collection site coordinates were extracted from the shapefile using GRASS-GIS software [28]; specifically, coordinates of Tanzanian collection sites were selected as the centroid of the collection district (Mwanza, Mbeya, Morogoro, Pemba, as described in [14]), and coordinates of the other states were selected as the centroids of those states.

### Computational performance

Algorithms were written in R, using the package 'ape' [29]. The runs were performed on the cluster IBM-BCX available at the Supercomputing Group of the CINECA Systems & Tecnologies Department between February and June 2010.

## Authors' contributions

FC implemented the computational model, carried out the simulations, and participated in the interpretation of the results. LB conceived the design of the study, implemented the computational model, and participated in the interpretation of the results. TLG conceived the design of the study and participated in the interpretation of the results. MG conceived the design of the study, participated in the interpretation of the results, and coordinated the participants' contributions. All authors participated in writing the manuscript and approved it.
